# Gut microbiome and dietary patterns in different Saudi populations and monkeys

**DOI:** 10.1038/srep32191

**Published:** 2016-08-31

**Authors:** Emmanouil Angelakis, Muhammad Yasir, Dipankar Bachar, Esam I. Azhar, Jean-Christophe Lagier, Fehmida Bibi, Asif A. Jiman-Fatani, Maha Alawi, Marwan A. Bakarman, Catherine Robert, Didier Raoult

**Affiliations:** 1 Unité de Recherche sur les Maladies Infectieuses et Tropicales Emergentes: URMITE CNRS-IRD 198 UMR 6236, Aix Marseille Université, Faculté de Médecine, 27 Bd Jean Moulin, 13385 Marseille, France; 2 Special Infectious Agents Unit, King Fahd Medical Research Center, King Abdulaziz University, Jeddah, Saudi Arabia; 3 Department of Medical Laboratory Technology, Faculty of Applied Medical Sciences, King Abdulaziz University, Jeddah, Saudi Arabia; 4 Department of Medical Microbiology and Parasitology, Faculty of Medicine, King Abdulaziz University, Jeddah, Saudi Arabia; 5 Infection Control Unit, King Abdulaziz University Hospital, King Abdulaziz University, Jeddah, Saudi Arabia; 6 Family and Community Medicine Department, Faculty of Medicine, King Abdulaziz University Rabigh, Saudi Arabia

## Abstract

Host genetics, environment, lifestyle and proximity between hosts strongly influence the composition of the gut microbiome. To investigate the association of dietary variables with the gut microbiota, we used 16S rDNA sequencing to test the fecal microbiome of Bedouins and urban Saudis and we compared it to the gut microbiome of baboons living in close contact with Bedouins and eating their leftovers. We also analyzed fermented dairy products commonly consumed by Bedouins in order to investigate their impact on the gut microbiome of this population. We found that the gut microbiomes of westernized urban Saudis had significantly lower richness and biodiversity than the traditional Bedouin population. The gut microbiomes of baboons were more similar to that of Bedouins compared to urban Saudis, probably due the dietary overlap between baboons and Bedouins. Moreover, we found clusters that were compositionally similar to clusters identified in humans and baboons, characterized by differences in *Acinetobacter, Turicibacter* and *Collinsella.* The fermented food presented significantly more bacteria genera common to the gut microbiome of Bedouins compared to urban Saudis. These results support the hypothesis that dietary habits influence the composition of the gut microbiome.

Recent advances in high-throughput technologies have revolutionized our knowledge of the gut microbiota and revealed a substantial diversity of the gut microbiota among individuals from different countries^1^,^2^. The existence of three enterotypes in the human gut microbiome that vary in species and functional composition was recently demonstrated using data that span several nations and continents^3^. Moreover, the characterization of the gut microbial communities in populations of great apes has provided insights into the origins of the human enterotypes^4^. We do not yet completely understand how the different environments and diets around the world have affected the microbial ecology of the human gut microbiota, and few studies have focused on the gut microbiomes of individuals exclusively eating locally^5^,^6^,^7^. Indeed, extant people living traditional lifestyles are especially under-studied, limited to hunter-gatherers from Tanzania^6^, rural communities in Burkina Faso^5^, in Malawi and Venezuela^8^ and recently in hunter-gatherer from Peru^7^. Comparative studies between non-industrialized rural communities and industrialized western communities have revealed gut microbiota adaptations to their respective lifestyles^9^. Unindustrialized rural societies are targets for understanding trends in human gut microbiota interactions as they rely less on antibiotics and often consume a greater breadth of unrefined seasonally available foods^10^. However, despite recent focus on rural societies, there remains an important gap in our knowledge of how local food influences their gut microbiome. Indeed diet plays a critical role in the gut microbiome and dietary habits can influence bacterial diversity^11^,^12^.

Saudi Arabia is considered one of the most rapidly growing economies in the world, where eating habits have completely changed in recent years. As a result, urban Saudis have drastically changed their lifestyle and food habits, with a very limited variety of foods and an absence of fruit and vegetables^13^. In contrast, rural Bedouins regularly consume vegetables, fruit and homemade fermented dairy products. As a result, because of the cultural, behavioral and ecological environment, we hypothesized that rural Bedouins harbor different microbiome profiles than those previously described in urban Saudis^14^. To test this hypothesis, we used high-throughput 16S ribosomal RNA (rRNA) gene amplicon sequencing to characterize their gut microbiota, and we compared it to those of urban Saudis. Moreover, to test the impact of specific dietary sources on the gut microbiome, we tested homemade fermented dairy products commonly consumed by Bedouins. In addition, the impact of proximity on the gut microbiome for several mammals has been previously reported, including chimpanzees^4^ wild apes^15^, dogs^16^, and gorillas^17^. This may be explained by a dietary overlap between the animal populations. Bedouins live in close contact with baboons, and there is a dietary overlap between human and primates. Specifically, primates commonly consume the leftover food of Bedouins (Supplementary Figure 1). To reinforce our hypothesis about the impact of food on the gut microbiome, we also tested the fecal microbiome of baboons living in sympatry with Bedouins.

## Results

### Composition of the gut microbiota of humans

**Figure 1:**
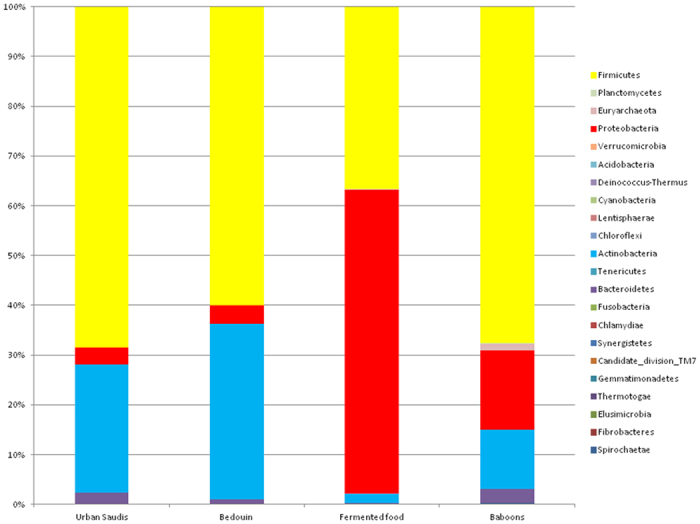
The relative abundance of the gut microbiota phyla among the groups tested.

We sequenced the V3-V4 region of 16S rDNA in fecal samples from 18 individuals from Jeddah living urban lifestyles and 10 Bedouins living rural lifestyles. The analysis of the high quality trimmed reads revealed that the gut microbiota of urban Saudis contained sequences from 8 different bacterial divisions/phyla, and most of the sequences belonged to *Firmicutes* and *Proteobacteria* (Fig. 1, Supplementary Table 1). The gut microbiota of Bedouins contained sequences from 10 different bacterial divisions/phyla, and most of the sequences belonged to *Firmicutes* and *Actinobacteria*. The relative abundance *Bacteroidetes* were significantly more common in the stools of urban Saudis compared to Bedouins (*p* = 0.01). Indeed, the relative abundance of *Bacteroides* has been associated positively in the past with diets rich in animal fat and protein^18^. The relative abundance of *Verrucomicrobia* were significantly more common in Bedouins compared to urban Saudis (*p* = 0.05). Finally, sequences of *Spirochaetae* and particulary *Treponema berlinense* and *Treponema succinifaciens* were only present in the gut microbiome of Bedouins. In the gut microbiome of urban Saudis, we detected 129 different genera whereas Bedouins had 157 different genera. All humans shared a core set of bacterial genera that was recovered from a majority of individuals from every sampled population. Indeed, we detected 74 genera in >50% of urban Saudis and 66 genera in >50% of Bedouins (Supplementary Figure 2). We then investigated the distribution of aerobic and facultative anaerobic genera residing in the gut microbiome of these groups using the taxonomic classification provided by 16S amplicon analysis. The difference in anaerobic genus counts revealed that urban Saudis had 78 different genera and Bedouins 76 different genera. As a result, significantly more different anaerobic genera existed in the gut microbiome of urban Saudis compared to Bedouins (Chi-square test; *p* = 0.04) but urban Saudis presented significantly more relative abundance of anaerobic genera compared to Bedouins (*p* < 0.001) (Supplementary Figure 3).

### Impact of Fermented food on gut microbiome of humans

Diet plays a critical role in the gut microbiota^14^,^19^, and we analyzed 6 fermented foods, called Lohoh in the local language, commonly consumed by Bedouins, in order to determine their impact on the gut microbiome of Bedouins. For their preparation, pearl millet flour is mixed with water in the ratio of 1:2 to make a dough (ajeen) and is incubated at 30 °C for 24 hours. Usually by this time the dough has a good consistency and sour taste. The dough was fermented by adding 5% inoculate (starter) from previously fermented dough to start each subsequent batch.

**Figure 2:**
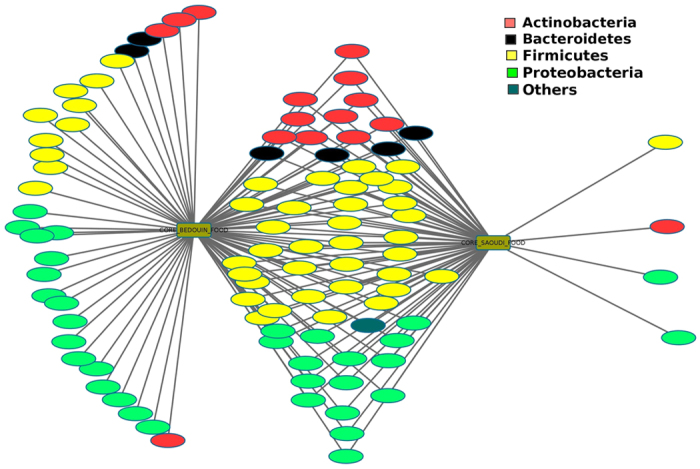
Network of bacterial genera present in fermented food and in the gut microbiome of urban Saudis and Bedouins.

We found that the food contained approximately 3 million 16S rRNA gene sequence reads, contained sequences from 11 different bacterial divisions/phyla, and most of the sequences belonged to *Firmicutes* and *Proteobacteria* (Fig. 1, Supplementary Table 1). We found 110 different genera, and most of the sequences belonged to *Halomonas, Lactobacillus* and *Shewanella*. In humans, 64 genera present in food also existed in the gut microbiome of both Bedouins and urban Saudis, whereas 3 genera present in food only existed in urban Saudis and 33 genera present in food only existed in the gut microbiome of Bedouins (Fig. 2, Supplementary Table 2). As a result, Bedouins presented significantly more bacteria genera present in fermented food compared to urban Saudis (*p* > 0.001). The genera *Acetobacter* and *Mycoplasma* present in food existed only in the gut microbiome of Bedouins. Finally, sequences of *Treponema berlinense* and *Treponema succinifaciens* were both presented in the food and the gut microbiome of Bedouins.

### Composition of the gut microbiota of baboons

We also sequenced 34 stools from baboons living in contact with Bedouins. The analysis of the high quality trimmed reads revealed that the gut microbiota of baboons contained sequences from 18 different bacterial divisions/phyla, and most of the sequences belonged to *Actinobacteria*, followed by *Firmicutes* (Fig. 1, Supplementary Table 1). *Fusobacteria* were only present in the stools of Bedouins and baboons. The relative abundance of *Bacteroidetes* were significantly more common in the stools of baboons compared to Bedouins (*p* = 0.02). Baboons presented significantly less relative abundance of *Actinobacteria* compared to Bedouins and urban Saudis (*p* = 0.002 and *p* > 0.001 respectively) and significantly less relative abundance of *Verrucomicrobia* compared to Bedouins (*p* = 0.008).

In the gut microbiome of baboons we detected 237 different genera (75 anaerobic) and 72 genera were presented in the gut microbiome of >50% baboons. Moreover we detected 32 genera that existed in the gut microbiome of >50% of both humans and baboons (Supplementary Figure 2). In animals, 12 genera present in food existed only in the gut microbiome of baboons, whereas 93 genera existed in the gut microbiome of both Bedouins and baboons. Significantly less different anaerobic genera existed in the gut microbiome of baboons compared to urban Saudis (Chi-square test; *p* = 0.0001), whereas Bedouins presented significantly more different anaerobic genera compared to baboons (Chi-square test; *p* = 0.001). Finally, baboons presented significantly less relative abundance of anaerobic genera compared to urban Saudi and Bedouin groups (*p* < 0.001 and *p* < 0.001 respectively) (Supplementary Figure 3).

### Gut microbiota proximity among humans and baboons

**Figure 3:**
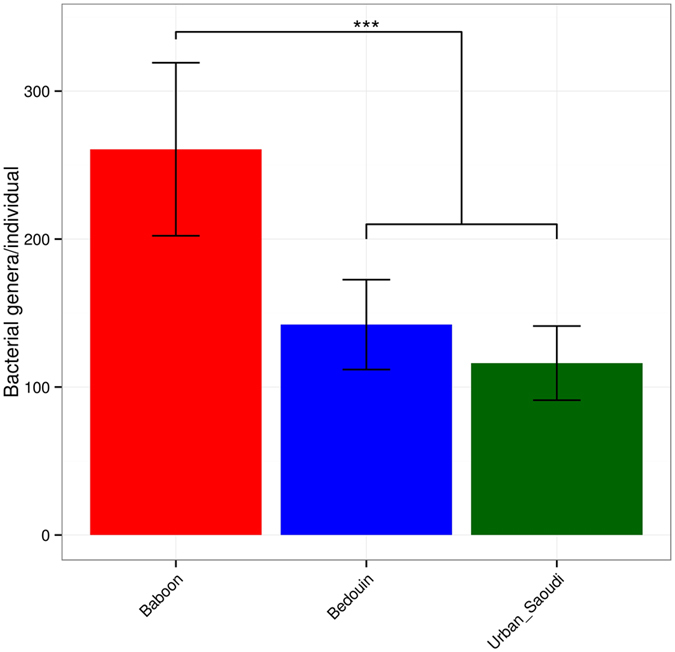
Diminished diversity in human and baboon gut microbiomes across populations. Mean numbers of observed bacterial genera per individual in baboons and in human populations at a sequencing depth of 20,000 reads. Error bars correspond to 95% CIs, and asterisks denote significant differences at *p* < 0.001.

Overall, 147 different genera from all sequence reads were identified, and based on genus-level Bray–Curtis dissimilarity (BCD), the microbiomes of baboons were more different than human microbiomes (*p* < 0.001) (Fig. 3). Moreover, Principle Coordinate Analysis (PCoA) of the overall composition of the genera communities between the groups revealed that the microbiome of baboons is closer to that of Bedouins than that of urban Saudis (Supplementary Figure 4). Comparing baboon and human microbiomes allowed us to infer the compositional changes that are most parsimoniously explained by the present-day variation among the microbiomes of human populations. The differences between microbiomes of urban Saudis and baboons was particularly evident in the first two principal coordinate axes of the pairwise beta diversities among samples at both the genus and 97% operational taxonomic unit (OTU).

The rarefaction with the measure chao1 shows that the samples from the baboon community are richer and more diverse than the other 3 groups (Supplementary Figure 5). Moreover, the microbial richness, estimated by the Chao1 index, and biodiversity, assessed by a nonparametric Shannon index for comparison among the groups, revealed that the gut microbiomes of baboons had significantly higher richness and biodiversity than both urban Saudis and Bedouins (Supplementary Figure 6). Moreover, we found that the gut microbiomes of westernized urban Saudis had significantly lower richness and biodiversity than the traditional Bedouin population.

### Enterotype-Like Clusters among humans and baboons

**Figure 4:**
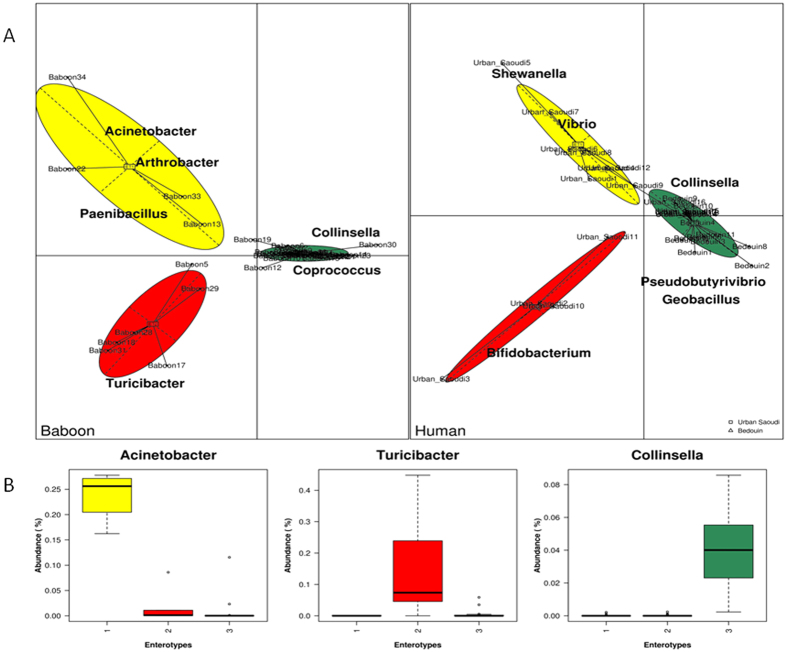
Identification of baboon enterotypes. (**A**) Assortment of gut microbial communities into enterotypes in baboons and humans. Shown are BCA visualizations of enterotypes (colored ellipses), as identified by PAM clustering, with black dots representing abundance distributions of bacterial genera from an individual host and numbered white rectangles marking the center of each enterotype. Panel (right) showing human gut enterotypes modified from Arumugam *et al*.^3^. Bacterial taxa uniquely overrepresented in the corresponding baboon and human enterotypes are listed. (**B**) Relative abundance of the three bacterial taxa that are principally responsible for the separation of baboon enterotypes. Shown are means, ranges and first and third quartiles.

To test for the presence of enterotypes in baboons, we performed a multidimensional cluster analysis and Principal Component Analysis (PCA) by employing the same clustering and cluster validation methods that Arumugam *et al*.^3^ used to identify the human enterotypes (Supplementary Figures 7 and 8). As in chimpanzees^4^, our analyses revealed that the gut microbiome of baboons based on their genus-level compositions into three distinct clusters (i.e., enterotypes) that do not significantly correlate with host age, genealogy or gender (Fig. 4A). The bacterial taxa identified by class analysis as contributing most significantly to each cluster were *Acinetobacter* in enterotype 3 baboons, *Turicibacter* in enterotype 2 baboons, and *Collinsella* in enterotype 1 baboons (Fig. 4B). For humans, the bacterial taxa *Shewanella* and *Vibrio* are significantly overrepresented in enterotype 3, *Bifidobacterium* in enterotype 2 and *Collinsella* in enterotype 1. Bacterial taxa *Collinsella* contributes significantly in both human and baboon enterotype 1. Despite the overall congruence between the human and baboon enterotypes, we found differences in the prevalence of several bacterial genera (Table 1). We found a broad correspondence between the baboon and human enterotypes, although several bacterial genera that were overrepresented in a baboon enterotype were not overrepresented in any of the human enterotypes. As a result, *Coprococcus* and *Marvinbryantia* were represented in baboon enterotype 1, *Turicibacter, Pedobacter* and *Vasilyevaea* were represented in baboon enterotype 2, and *Acinetobacter, Paenibacillus, Arthrobacter, Pectobacterium, Desemzia* and *Pseudomonas* were represented in baboon enterotype 3, but these genera did not contribute significantly to the human enterotypes (Tables 1.2 and Supplementary Table 2).

## Discussion

In this study, the comparison of the gut microbiomes of populations of humans and baboons, as well as dairy products commonly consumed by Bedouins, provides us with important information about the impact of dietary habits on gut microbiomes. In particular, we have demonstrated that the similarity in the microbiota of cohabiting individuals extends beyond human-to-human relationships and to animal-to-human relationships. We also provide evidence that baboons possess enterotypes that are compositionally similar to those observed within human populations, and that food has an important impact on the composition of the gut microbiome. All our samples were collected under similar conditions and they were kept frozen under sterile conditions at −80 °C, eliminating the possibility of contamination. In addition, before analyses, we verified that all of our samples had a good DNA load, and 16S rRNA sequencing-based studies targeting the V3-V4 region have been commonly used for the determination of the gut microbiome. Finally, our analysis for the presence of enterotypes was previously validated in humans^3^ and in animals^4^.

Previous reports have indicated that Western populations have less microbial richness than non-Western populations^5^. Our analyses of microbial richness yielded similar results, as Bedouins regularly consumed vegetables, fruit, chicken, dairy products, fermented food and rice. Vegetables and fruit were part of the daily diet in Bedouin populations compared to urban Saudis, who reported vegetable and fruit consumption only 1–2 times per week. Indeed, the urban population in Saudi Arabia has shifted away from traditional food to Western diets^13^. Most of the participants reported that they consumed a lot of snacks and fast foods, such as shawarma, hamburger, pizza and fried chicken. Regular consumption of junk-food snacks, eating away from home and abundant use of carbonated beverages in the Saudi population has already been reported^13^. A high-fiber diet has been associated with an enrichment of the microbiome^5^, and differences associated with diet were found in the gut microbiota of different populations^6^,^8^,^9^. Moreover, the gut microbiota can rapidly respond to an altered diet, potentially facilitating the diversity of human dietary lifestyles^20^,^21^. It was previously proposed that microbial diversity in the human microbiomes has decreased during human evolution^4^, and that recent lifestyle changes in humans have depleted the human microbiome of microbial diversity that was present in ancestors living in the wild^22^.

In this work, we found that the gut microbiome of Bedouins was closer to that of baboons than that of urban Saudis. This may possibly be explained by a dietary overlap with Bedouins. In fact, baboons rely on the leftover food from the local population dumped by the municipality or the food shared by people with them. People bring food like khubus (a type of local bread) and fruit for baboons. Moreover, baboons raid local farms and crops for their food. This is reinforced by the fact that we found that food affects the gut microbiome, because we found significantly more bacteria genera present in fermented food in the microbiome of Bedouins than in urban Saudis who do not consume these products. Indeed, the average consumption of fermented food and dairy products per week is much higher in the Bedouins compared to urban Saudis, and it is estimated that Bedouins consume these fermented foods on average 5–8 times per week. Moreover, the types of dietary pattern are almost the same in Bedouin populations. However, a limitation of our study was that we did not measure the amount of each food consumed among the populations. Moreover the use of bread to avoid baboon’s aggression did not modify their gut microbiota as stools were collected the same time. Baboons and Bedouins presented almost the same bacteria genera present in fermented food in their gut microbiome, indicating that the effect of dietary choices is a very important factor affecting the gut microbiome.

As previously reported for humans^3^,^18^ and animals^4^,^23^, we have demonstrated the existence of three enterotypes in the human and baboon gut microbiome. Long-term diets were previously correlated with enterotype status, where individuals with greater animal fat and protein intake were more likely to present the Bacteroides-dominated enterotype compared with those with more carbohydrate intake, associated with a Prevotella-dominated enterotype^18^,^23^. Moeller *et al*.^4^ found that chimpanzees similarly presented three enterotypes, contributed by *Faecalibacterium, Lachnospiraceae* and *Bulleidia* respectively. In contrast, the enterotypes of baboons were contributed by *Acinetobacter, Turicibacter* and *Collinsella.* Despite marked differences among the microbiomes of humans and baboons, we found that there is a set of bacterial taxa shared across host populations. Gender has been previously correlated with modifications of the gut microbiota^24^, but a limitation of our study was that, due to ethical reasons, we tested only male individuals from Jeddah living urban lifestyles. Although the ecological/cultural divergence between humans can explain the differences among their microbiomes, the relative roles of genetic divergence between host species in generating the differences between their microbiomes remain unclear.

In conclusion, we have shown that baboons possess enterotypes that are compositionally similar to those observed in human populations, and we confirmed that Western diet populations have less microbial richness than traditional populations. The dietary overlap between baboons and Bedouins probably explains why these populations present similarities in their gut microbiome compared to urban individuals. Moreover, the fact that the fermented food presented significantly more bacteria genera common to the gut microbiome of Bedouins compared to urban Saudis shows the impact of diet on the gut microbiome. We point out the importance of more intensive research in the future to understand the impact of diet-microbiome interactions.

### Online Materials

#### Subject selection criteria

This study protocol was approved by the Ethics Committee of King Abdul Aziz University under agreement No. (014-CEGMR-2-ETH-P), and methods were carried out in accordance with the approved guidelines. We tested only male volunteers from Jeddah living urban lifestyles and 8 male and 2 female Bedouins living rural lifestyles. The data (date of birth, weight, height, antibiotic use, dietary pattern and significant changes in diet) were recorded using a standardized questionnaire. Exclusion criteria included individuals under 18 years of age, a past history of colon cancer, inflammatory bowel disease, acute or chronic diarrhea in the previous 8 weeks and treatment with an antibiotic in the 3 months before fecal sampling. All patients gave written informed consent. We also collected baboon stool samples from the Taif region (21°26′N 40°21′E) located in the western province of Makkah. It is estimated that approximately 2,000 baboons live in this area and feed on garbage. Indeed, the baboons have become dependent on humans for food. No experimentation was conducted on baboons, as fecal samples were collected from the soil. No other permit was required, as this research was non-invasive work, and the collection of the samples did not disrupt the wild fauna. We fed baboons with bread to avoid their aggression and followed the herd of adult baboons for sample collection. Only little bread was used per herd of the baboon while stool samples were collected at the same time. We followed a different herd at seven different locations in that area to maximize samples from different baboons. We collected fresh stool samples with sterile spoons, avoiding the soil particles. Troops of baboons consisted of both male and female baboons; however, gender information was not possible to be collected for the sampled baboon. All stool samples were stored at −80 °C.

In addition, we collected different types of millet-fermented food called Lohoh that is commonly consumed by Bedouins. This food is prepared from different types of millet flour, such as Pearl millet flour, Injera millet flour and whole grain flour. These samples were collected in a sterile container from the Bedouin people at the time of stool sample collection and were stored at −80 °C.

#### Extraction of DNA from stool samples and 16S rRNA sequencing using MiSeQ technology

Fecal DNA was extracted from samples using the NucleoSpin^®^ Tissue Mini Kit (Macherey Nagel, Hoerdt, France) according to a previously described protocol^25^. Samples were sequenced for 16S rRNA sequencing using MiSeq technology as described by the manufacturer’s Guide 15044223- B from the MiSeq procedure. Briefly, PCR amplified templates from genomic DNA using the surrounding conserved region V3-V4 primers with overhang adapters (FwOvAd_341F TCGTCGGCAGCGTCAGATGTGTATAAGAGACAGCCTACGGGNGGCWGCAG;ReOvAd_785RGTCTCGTGGGCTCGGAGATGTGTATAAGAGACAGGACTACHVGGGTATCTAATCC). Samples were amplified individually for the 16S “V3-V4” regions by Taq Phusion and visualized on the Caliper LabchipII device by a DNA 1K LabChip. After purification on AMPure beads, the concentrations were measured using high sensitivity Qubit technology. Using a subsequent limited cycle PCR on 1 ng of each PCR product, Illumina sequencing adapters and dual-index barcodes were added to each amplicon. After purification on AMPure beads, the libraries were then normalized according to the Nextera XT protocol. The 96 multiplexed samples were pooled into a single library for sequencing on the MiSeq. The pooled library containing indexed amplicons was loaded onto the reagent cartridge and then onto the instrument along with the flow cell. Automated cluster generation and paired-end sequencing with dual index reads was performed in a single 39-hour run in a 2 × 250 bp. On the instrument, the global cluster density and the global passed filter per flowcell were generated. MiSeq Reporter software determined the percentage of indexing and cluster passed filter (PF) for each amplicon or library. The raw data were configured in fastq files for R1 and R2 reads.

#### Data processing: Filtering the reads, dereplication and clustering

The paired end reads of corresponding raw fastq files were assembled into contigs by using FLASH^26^, which gave a total of 14,428,091 assembled sequences. The high quality sequences were then selected for the next steps of analysis by considering only those sequences which contain both the primers (forward and reverse). In the following filtering steps the sequences containing N were removed. Sequences with a length shorter than 200 nts were removed and the sequences longer than 500 nts were trimmed. Both the forward and reverse primers were also removed from each of the sequences. An additional filtering step was applied to remove the chimeric sequences by using chimera slayer from QIIME^27^. After these filtering steps, a total of 13,085,792 sequences remained. Strict dereplication (clustering of duplicate sequences) was performed on these filtered sequences, and they were then sorted by decreasing abundance^28^. Next, clustering was performed with 97% identity and the OTUs (representative sequence of each cluster) were extracted^28^. These OTUs represented a total of 11,989,797 sequences. The above filtering steps and OTU extractions were performed in QIIME^27^. All the raw sequences of fastq files have been submitted to EMBL-EBI^29^ with the accession number PRJEB9815.

#### Building the reference database

We downloaded the Silva SSU and LSU database^30^ of release 119 from the Silva website and a local database of predicted amplicon sequences was built from it, after extracting the Siva reference sequences containing both the forward and reverse primers and by allowing 3 differences between each primer and the sequence^31^. Lastly, we had our local reference database containing a total of 456,714 well-annotated sequences.

#### Taxonomic Assignments

The OTUs were then searched against our reference database Silva 199 by using blast^32^. The 100 best matches above 80% identity (similarity) with each of the OTUs were extracted from the reference database and were sorted with respect to their decreasing percentage of similarity. Thus, the best hits with the highest percentage of similarity (by also considering all the hits within 0.5% similarity of the best hits) with the OTUs were then considered for taxonomic assignments, and taxonomy to the lowest rank was obtained by applying majority voting^28^.

#### Database of obligate anaerobes

We conducted a bacterial oxygen tolerance database based on the literature (available online at http://www.mediterranee-infection.com/article.php?laref=374). Each phylotype was assigned as ‘obligate anaerobe’, ‘aerotolerant’ or ‘unknown’ according to oxygen tolerance.

#### Statistical Analysis

We calculated the richness and biodiversity index of the OTUs by using the QIIME software package^33^. We estimated richness using the Chao1 index and diversity by the non-parametric Shannon formula^34^. Non-parametric Kruskal-Wallis along with Mann-Whitney analyses were performed to identify significantly different bacterial taxa in the study participants. Linear discriminant analysis was performed using Lefse^35^ with normalized option. We used QIIME for rarefaction and Principal Coordinate Analysis^27^. For PCoA, first we normalized the data at 20,000 sequences for each sample and calculated the weighted-unifrac distance^27^. We performed the Adonis^36^ test using this weighted-unifrac distance. PCoA was plotted using the weighted-unifrac distance^27^. We performed enterotype analyses (Supplementary Figures 7 and 8) by employing the PAM clustering and silhouette cluster validation technique, as described in the paper of Arumugam *et al*.^3^ using the R statistical software package. For enterotype analysis, the Jensen-Shannon Distance (JSD) distance of genus abundance was used for clustering^3^, the Calinski-Harabasz (CH) Index^37^ was used to assess the optimal number of clusters and the Silhouette coefficient^38^ was used for cluster validation. At the final step of enterotype analysis, the Principal Component Analysis (PCA) and Between Class Analysis (BCA) were performed and the results were plotted^3^. Other statistical analyses were performed using SPSS (IBM Corp. Released 2011. IBM SPSS Statistics for Windows, Version 20.0. Armonk, NY: IBM Corp.).

## Additional Information

**How to cite this article**: Angelakis, E. *et al*. Gut microbiome and dietary patterns in different Saudi populations and monkeys. *Sci. Rep.* **6**, 32191; doi: 10.1038/srep32191 (2016).

## Supplementary Material

Supplementary Information

## Figures and Tables

**Table 1:** Frequencies of bacterial taxa overrepresented within each baboon enterotype.

Taxa in Baboons	Frequency in		
	Enterotype1	Enterotype2	Enterotype3
**Enterotype 1**			
*Collinsella*	>0.0001	>0.0001	0.02
*Coprococcus*	>0.0001	0.0005	0.01
*Blautia*	0.0006	0.002	0.08
*Marvinbryantia*	>0.0001	0.0002	0.01
**Enterotype 2**			
*Turicibacter*	>0.0001	0.005	0.0003
*Pedobacter*	0.0004	0.02	0.0002
*Vasilyevaea*	>0.0001	0.001	0
**Enterotype 3**			
*Acinetobacter*	0.2	0.03	0.04
*Paenibacillus*	0.008	0.0007	0.0002
*Arthrobacter*	0.12	0.07	0.003
*Pectobacterium*	0.002	>0.0001	>0.0001
*Desemzia*	0.002	>0.0001	0.0001
*Pseudomonas*	0.02	0.001	0.001

**Table 2:** Frequencies of bacterial taxa overrepresented within each human enterotype.

Taxa in Humans	Frequency in		
	Enterotype1	Enterotype2	Enterotype3
**Enterotype 1**			
*Pseudobutyrivibrio*	0.06	0.003	0.003
*Collinsella*	0.05	0.01	0.03
*Papillibacter*	>0.0001	0	>0.0001
*Geobacillus*	>0.0001	>0.0001	>0.0001
**Enterotype 2**			
*Bifidobacterium*	0.06	0,1	0.04
*Blautia*	0.05	0.1	0.02
*Streptococcus*	0.005	0.07	0.02
**Enterotype 3**			
*Shewanella*	0	0	0.003
*Vibrio*	0	0	>0.0001
*Brochothrix*	0	0	>0.0001
