# Supplementary material for: Gut microbiome and dietary patterns in different Saudi populations and monkeys
Source: Sci Rep. 2016 Aug 31;6:32191. doi: 10.1038/srep32191 (PMC5006041; doi:10.1038/srep32191)
Supplement: Supplementary Information [file srep32191-s1.pdf]

Running heading: Gut microbiota of Saudis

Gut microbiome and dietary patterns in different Saudi populations and monkeys

Emmanouil Angelakis [1], Muhammad Yasir[2], Dipankar Bachar[1], Esam I. Azhar[2,3], Jean-Christophe Lagier[1], Fehmida Bibi[2], Asif A. Jiman-Fatani[4], Maha Alawi[4,5],Marwan A. Bakarman[6], Catherine Robert[1], Didier Raoult[1,2*]

[1]Unité de Recherche sur les Maladies Infectieuses et Tropicales Emergentes: URMITE CNRS-IRD 198 UMR 6236, Aix Marseille Université, Faculté de Médecine, 27 Bd Jean Moulin, 13385 Marseille, France

[2]Special Infectious Agents Unit, King Fahd Medical Research Center, King Abdulaziz University, Jeddah, Saudi Arabia

[3]Department of Medical Laboratory Technology, Faculty of Applied Medical Sciences, King Abdulaziz University, Jeddah, Saudi Arabia

[4]Department of Medical Microbiology and Parasitology, Faculty of Medicine, King Abdulaziz University, Jeddah, Saudi Arabia

[5]Infection Control Unit, King Abdulaziz University Hospital, King Abdulaziz University, Jeddah, Saudi Arabia

[6]Family and Community Medicine Department, Faculty of Medicine, King Abdulaziz University Rabigh, Saudi Arabia

*Corresponding author. Didier.raoult@gmail.com

Supplementary figure 1. Baboons eating the humans rests

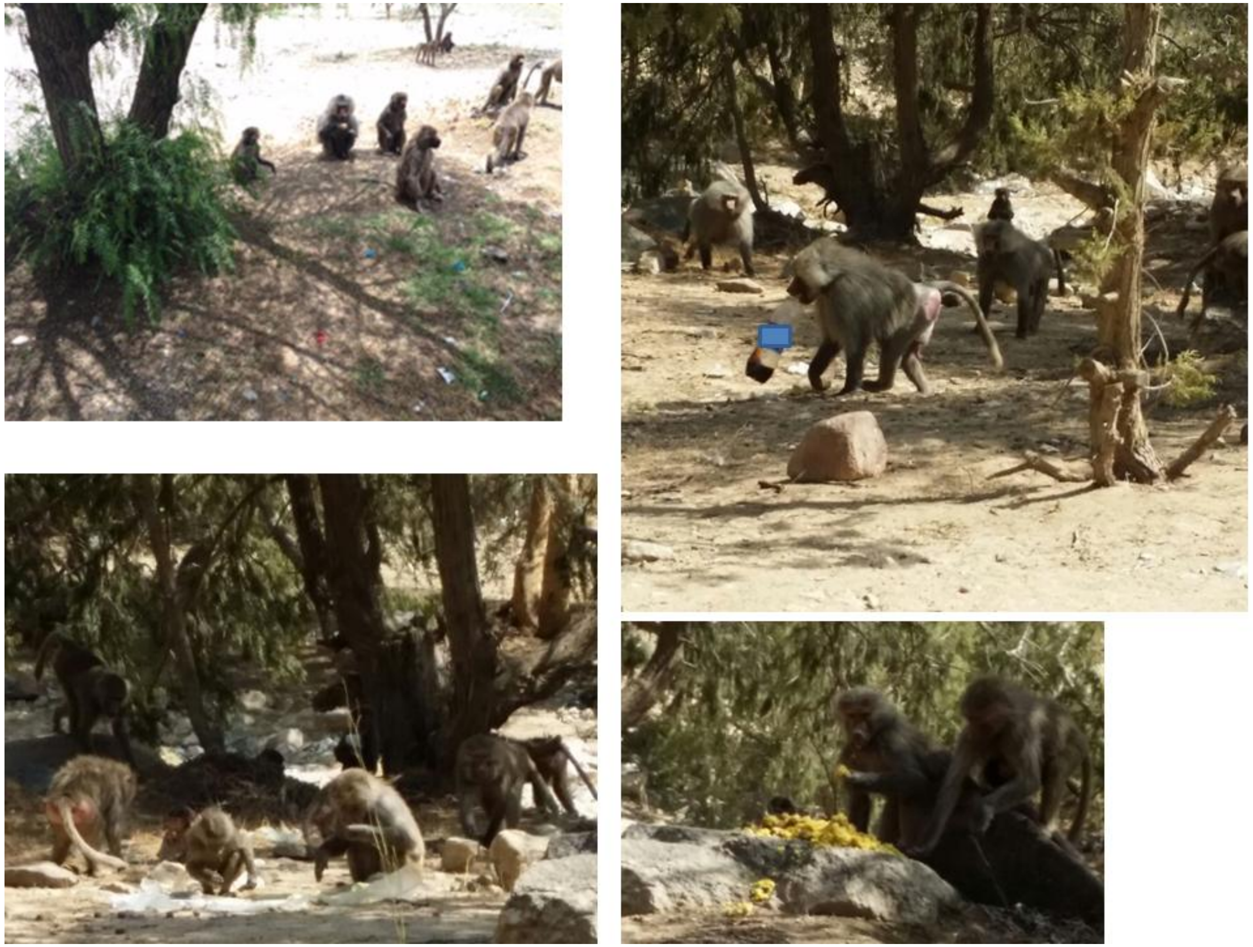

Supplementary figure 2. Venn diagram using the core species (species present at least 50 % of sample group) in each group.

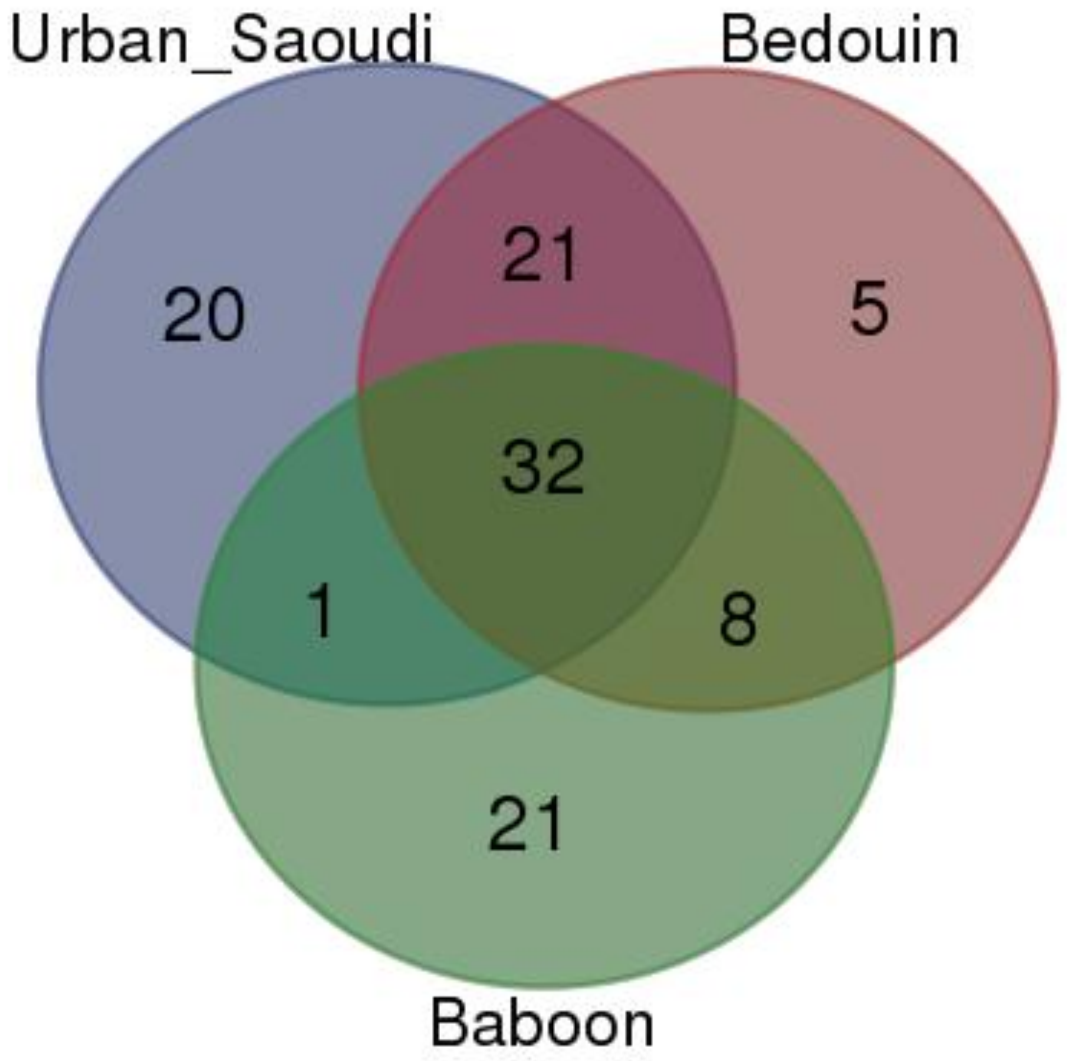

Supplementary Figure 3. Relative abundance of anaerobic and aerobic genera in the microbiome of urban Saudis, Bedouins and baboons.

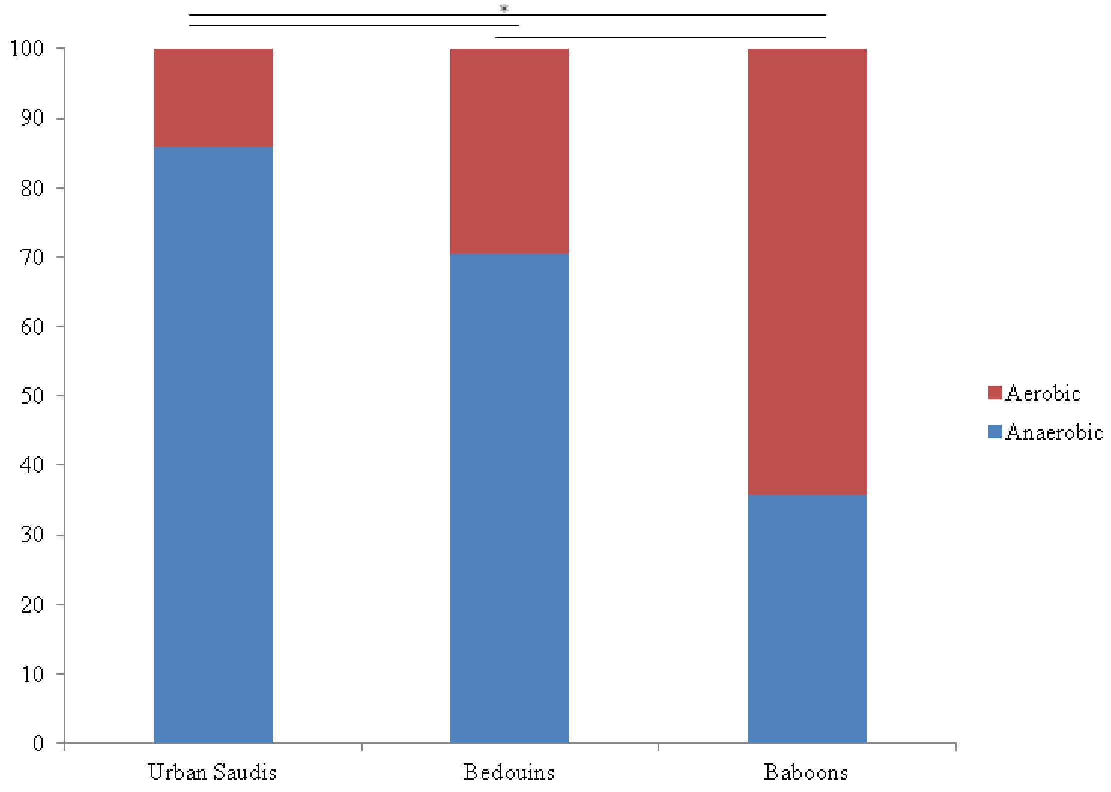

Supplementary figure 4. Principle Coordinate analysis of the overall composition of the genera communities

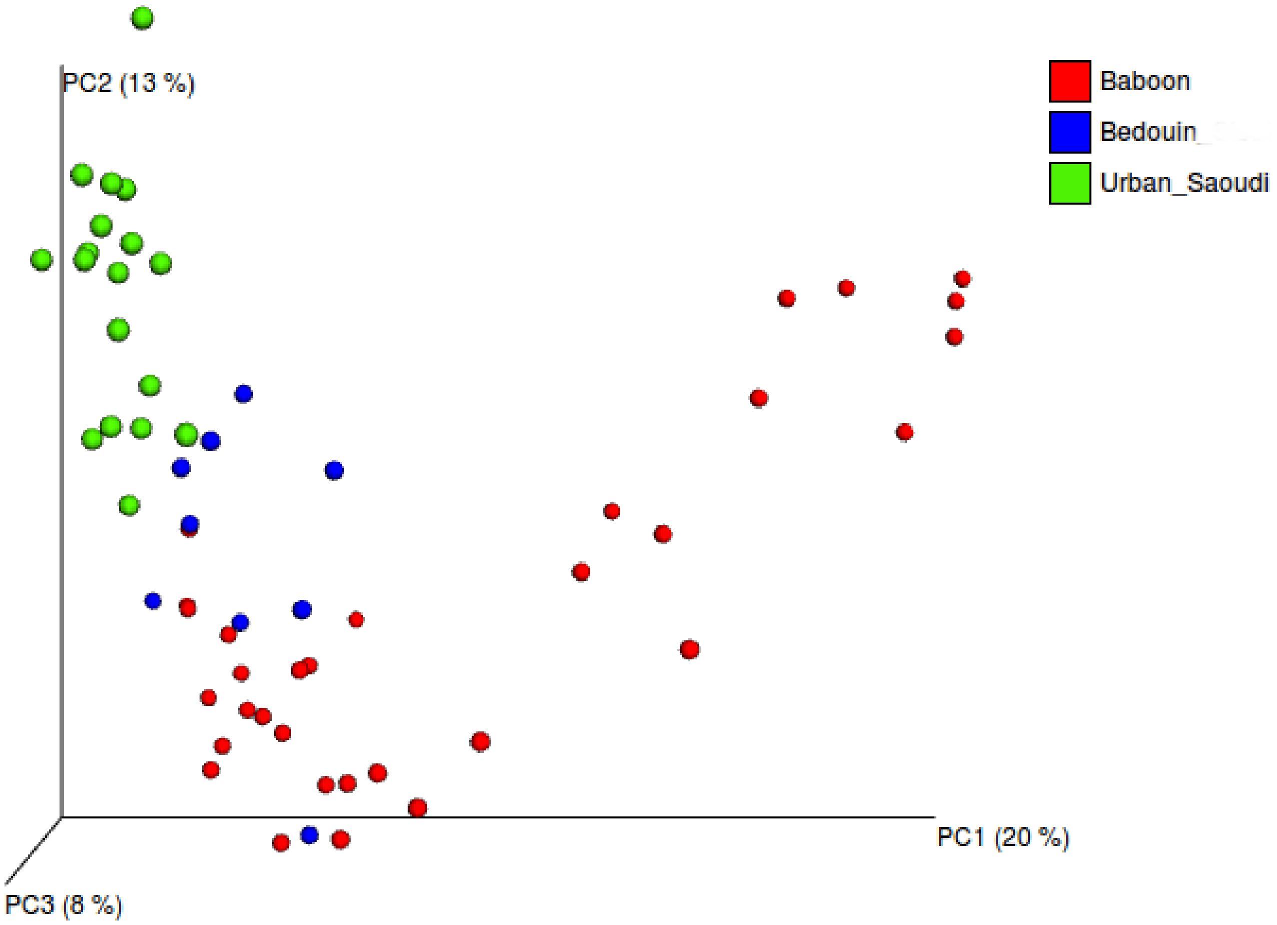

Supplementary figure 5. Rarefaction curve using Chao1

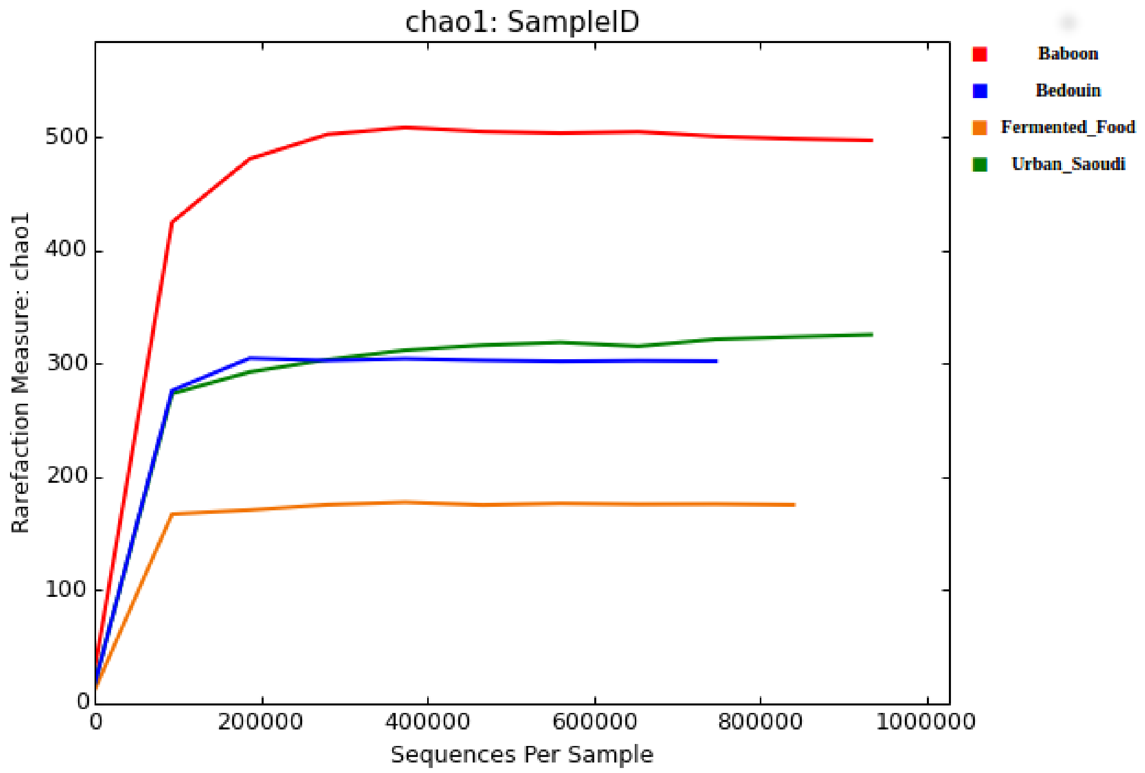

Supplementary figure 6. OTUs at different cutoffs

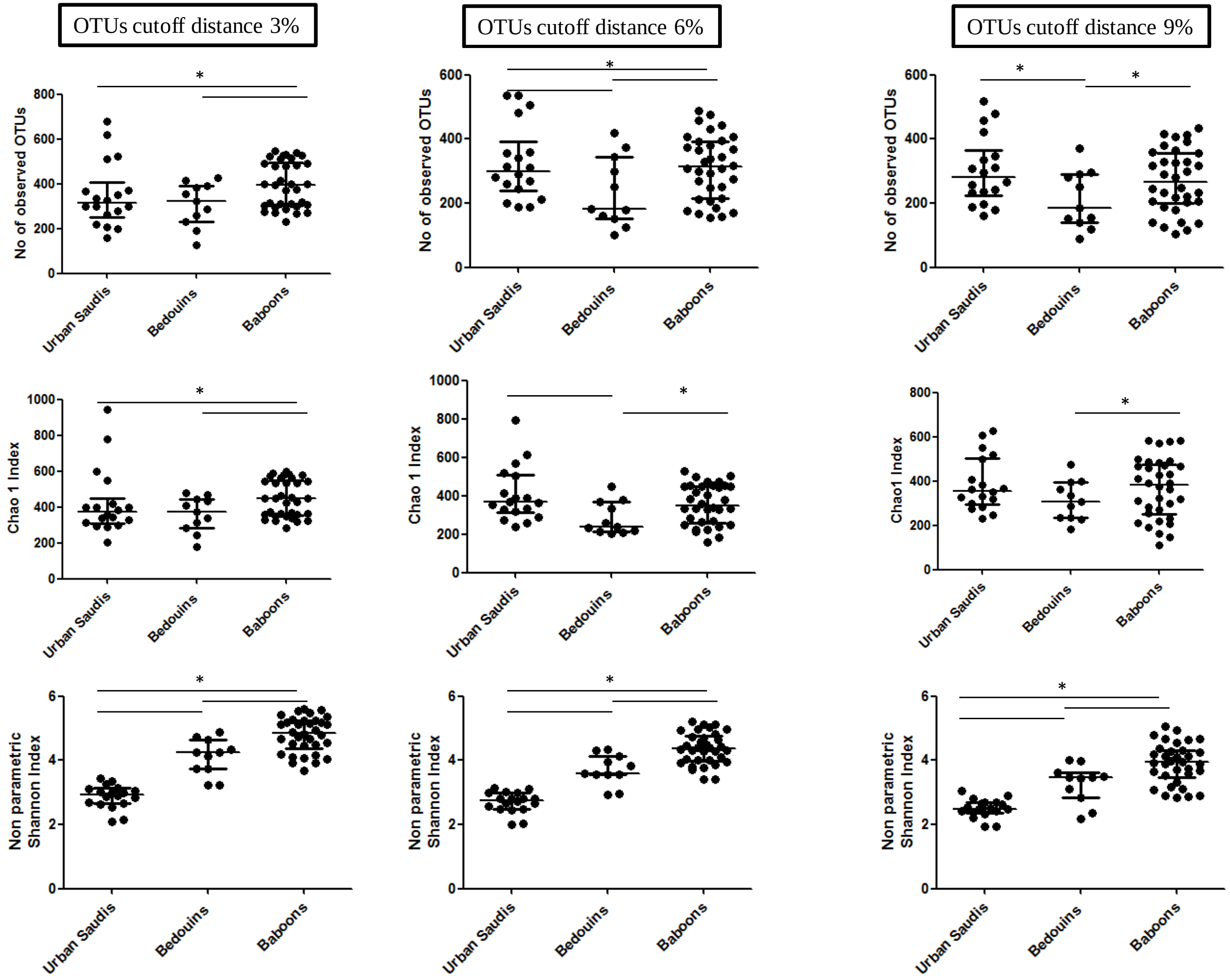

Supplementary Figure 7. Calinski-Harabasz Index showing optimal number of clusters in Enterotype analysis for the group Human.

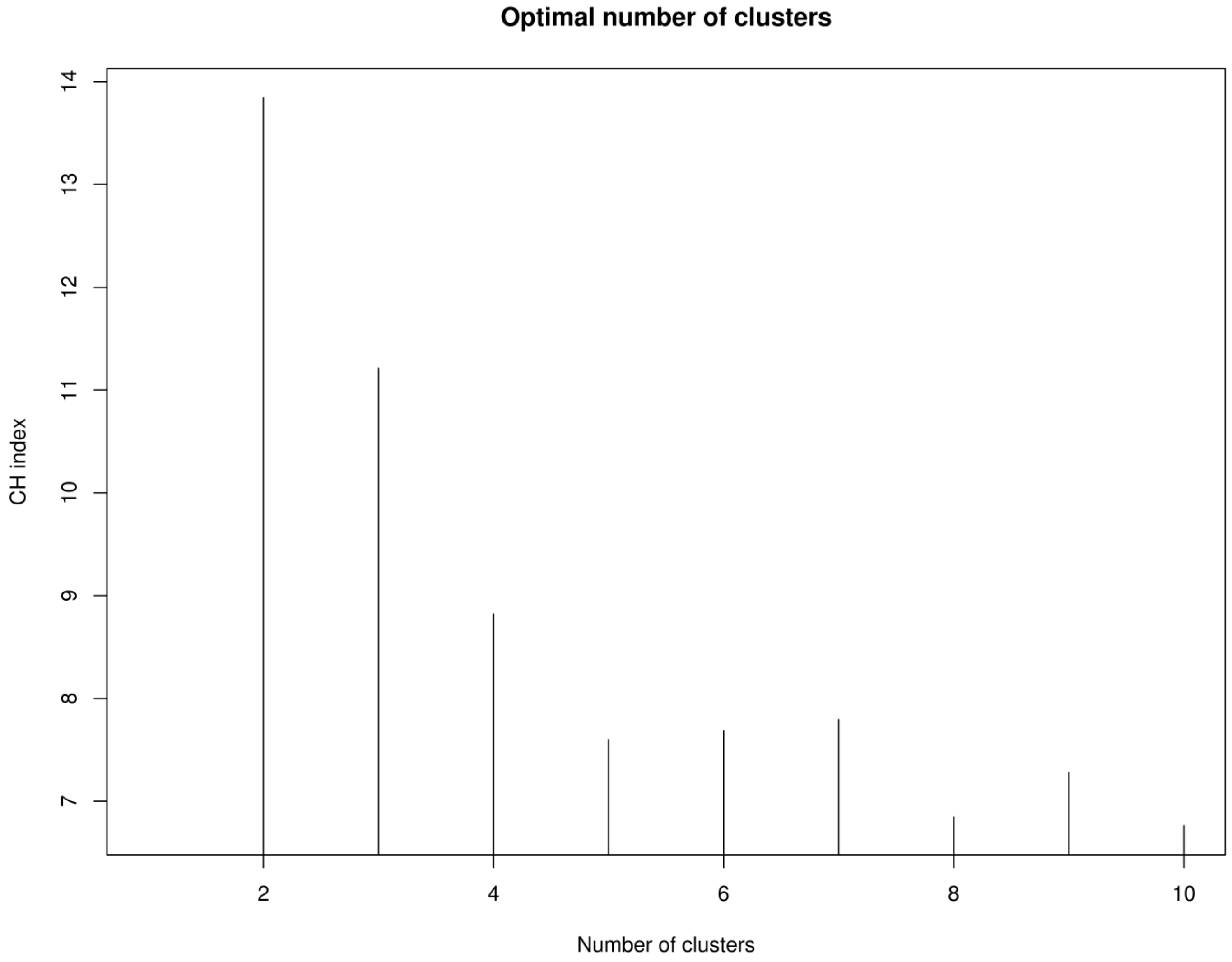

Supplementary Figure 8. Calinski-Harabasz Index showing optimal number of clusters in Enterotype analysis for the group Baboon.

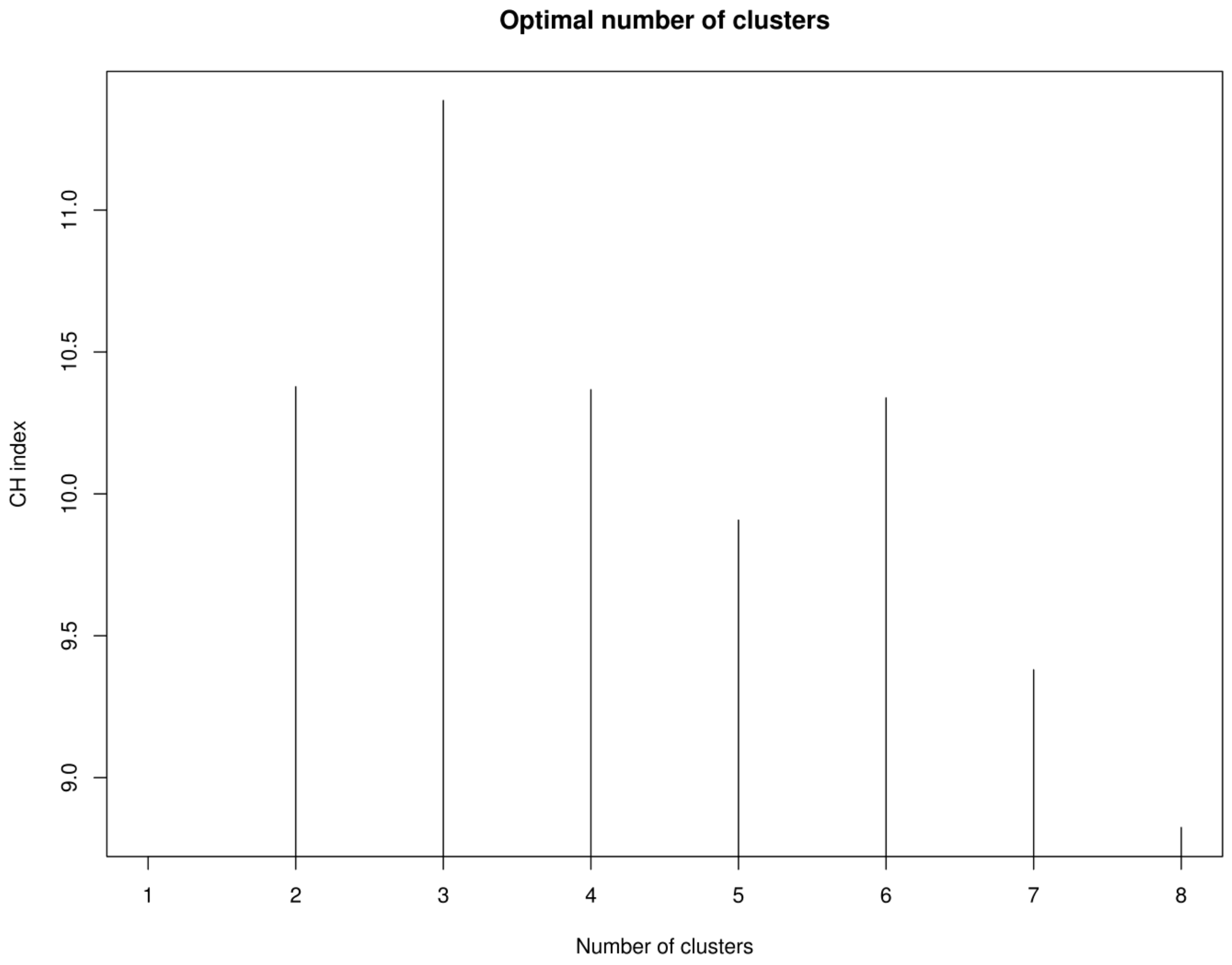

Supplementary table 1. Absolute numbers of the different bacterial divisions/phyla

**Phylum**	**Bedouin**	**Fermented Food**	**Baboon**	**Urban Saudis**
Spirochaetae	602	75	6,394	0
Fibrobacteres	0	0	138	0
Elusimicrobia	0	0	122	0
Gemmatimonadetes	0	0	68	0
Candidate_division_TM7	917	0	908	0
Fusobacteria	69	485	32	8
Bacteroidetes	16,966	2,825	95,548	104,617
Tenericutes	15	3	1,453	0
Actinobacteria	495,166	20,290	392,292	1,446,351
Chloroflexi	0	0	114	0
Lentisphaerae	0	0	860	20
Cyanobacteria	0	157	34	0
Deinococcus-Thermus	0	490	202	0
Verrucomicrobia	76,145	616	628	2,026
Proteobacteria	146,507	710,274	534,011	176,161
Euryarchaeota	48,687	802	40,934	129
Planctomycetes	0	0	5,593	0
Firmicutes	1,227,258	426,020	2,255,924	3,423,266

Supplementary table 2. Bacteria genera presented in food and in the gut microbiome of baboons, Bedouin and urban Saudis

**Bacteria genera**	**Urban Saudis**	**Bedouins**	**Baboons**
Pseudobutyrivibrio	1	1	1
Veillonella	1	1	1
Solobacterium	1	1	1
Escherichia-Shigella	1	1	1
Howardella	1	1	1
Ruminococcus	1	1	1
Enterobacter	1	1	1
Collinsella	1	1	1
Coprococcus	1	1	1
Clostridium sensu stricto1	1	1	1
Acinetobacter	1	1	1
Neisseria	1	1	1
Blautia	1	1	1
Succinivibrio	1	1	1
Undibacterium	1	1	1
Lactobacillus	1	1	1
Fusobacterium	1	1	1
Streptococcus	1	1	1
Dialister	1	1	1
Citrobacter	1	1	1
Anaerostipes	1	1	1
Bacillus	1	1	1
Marvinbryantia	1	1	1
Sphingomonas	1	1	1
Lactococcus	1	1	1
Granulicatella	1	1	1
Paenibacillus	1	1	1

Klebsiella	1	1	1
Micrococcus	1	1	1
Staphylococcus	1	1	1
Roseburia	1	1	1
Nocardioides	1	1	1
Enterococcus	1	1	1
Haemophilus	1	1	1
Phascolarctobacterium	1	1	1
Faecalibacterium	1	1	1
Corynebacterium	1	1	1
Methanobrevibacter	1	1	1
Pediococcus	1	1	1
Catenibacterium	1	1	1
Mogibacterium	1	1	1
Dorea	1	1	1
Rothia	1	1	1
Paracoccus	1	1	1
Propionibacterium	1	1	1
Tepidiphilus	1	1	1
Bifidobacterium	1	1	1
Oribacterium	1	1	1
Subdoligranulum	1	1	1
Akkermansia	1	1	1
Mitsuokella	1	1	1
Turicibacter	1	1	1
Enterorhabdus	1	1	1
Weissella	1	1	1
Alistipes	1	1	1
Alloprevotella	1	1	1
Bacteroides	1	1	1

Prevotella	1	1	1
Gemella	1	1	1
Actinomyces	1	1	1
Leuconostoc	1	1	1
Pseudomonas	1	1	1
Kocuria	1	1	1
Stomatobaculum	1		
Nocardiopsis	1		
Acetobacter		1	
Mycoplasma		1	
Rubellimicrobium			1
Rahnella			1
Curtobacterium			1
Dietzia			1
Macrococcus			1
Microbacterium			1
Rummeliibacillus			1
Erwinia			1
Paucimonas			1
Novosphingobium			1
Solibacillus			1
Leptotrichia			1
Parvimonas	1	1	
Photobacterium		1	1
Delftia		1	1
Marinomonas		1	1
Tsukamurella		1	1
Massilia		1	1
Porphyromonas		1	1
Brochothrix		1	1

Vibrio		1	1
Psychrobacter		1	1
Halomonas		1	1
Treponema		1	1
Planococcus		1	1
Shewanella		1	1
Sporosarcina		1	1
Enhydrobacter		1	1
Sinomonas		1	1
Psychromonas		1	1
Arthrobacter		1	1
Anaerovibrio		1	1
Exiguobacterium		1	1
Chryseomicrobium		1	1
Sarcina		1	1
Burkholderia		1	1
Virgibacillus		1	1
Butyrivibrio		1	1
Pseudonocardia		1	1
RC9 gut group		1	1
Gluconobacter		1	1
Planomicrobium		1	1
Pantoea		1	1
	66	96	105
